# Drug Resistance in Tuberculous Lymphadenitis: Molecular Characterization

**DOI:** 10.1155/2023/3291538

**Published:** 2023-03-29

**Authors:** Gebeyehu Assefa, Kassu Desta, Shambel Araya, Selfu Girma, Elena Hailu, Adane Mihret, Tsegaye Hailu, Melaku Tilahun, Getu Diriba, Biniyam Dagne, Abay Atnafu, Nigatu Endalafer, Adugna Abera, Shiferaw Bekele, Yordanos Mengistu, Kidist Bobosha, Abraham Aseffa

**Affiliations:** ^1^Armauer Hansen Research Institute, AHRI, Addis Ababa, Ethiopia; ^2^Department of Medical Laboratory Sciences, College of Health Sciences, Addis Ababa University, Addis Ababa, Ethiopia; ^3^Ethiopian Public Health Institute, EPHI, Addis Ababa, Ethiopia; ^4^Haramaya University, Harar, Ethiopia

## Abstract

**Background:**

Drug-resistant tuberculosis (TB) epidemic in high-TB-incidence countries, particularly Ethiopia, remains a significant challenge. As a result, we investigated the drug resistance, common gene mutation, and molecular characterization of mycobacterial isolates from patients with suspected tuberculous lymphadenitis (TBLN). *Methodology*. A cross-sectional study of 218 FNA samples from TBLN patients inoculated on Lowenstein-Jensen media was carried out. The culture isolates were identified as MTB by polymerase chain reaction (PCR) and the difference-9 (RD9) test region. In addition, the GenoType MTBDR*plus* assay tested the first and second-line MTB drugs, and the spoligotyping strain-dependent polymorphism test was determined.

**Results:**

Among the 50 culture-positive isolates, 14% (7/50) had drug resistance caused by a gene mutation. Out of these, 4 (8%) isolates were mono-resistant to isoniazid drug, which is caused by a gene mutation in *katG* in the region of interrogated at codon 315 in the amino acid sequence of S315T1, and 3 (6%) isolates were resistant to both rifampicin and isoniazid drugs. The mutation was observed for *katG* (at codon 315 with a change in the sequence of amino acid S315T) and *rpoB* (at codon 530–533 with a change in the sequence of amino acid S531L (S450L)) genes. The most prevalent spoligotypes were orphan and SIT53 strains.

**Conclusion:**

The predominance of INH mono-resistance poses a critical risk for the potential development of MDR-TB, as INH mono-resistance is a typical pathway to the occurrence of MDR-TB. The orphan and SIT53 (T) strains were the most common in the study area, and a drug-resistant strain caused by a common gene mutation could indicate the transmission of clonal-resistant strains in the community.

## 1. Background

Tuberculosis (TB), caused by *Mycobacterium tuberculosis*, remains a global health concern causing significant mortality and morbidity, particularly in developing countries. The World Health Organization reported that 10 million people were affected, with 1.3 million deaths and 214,000 people living with human immunodeficiency virus (HIV) in 2020 [1]. The percentage of HIV-positive TB patients worldwide is 8%. This percentage approaches 50% in parts of Southern Africa, where it is most significant in the African continent [1]. In addition, TB accounts for approximately 14% of all acquired immunodeficiency syndrome- (AIDS-) related deaths [1]. The most commonly focused form of TB in surveillance is pulmonary tuberculosis (PTB), which affects the lungs as the primary organ, and this could lead to a depreciation of extrapulmonary tuberculosis (EPTB) [2]. EPTB was responsible for 16% of the 7.1 million TB incident cases recorded globally in 2019 [3].

Multidrug-resistant TB (MDR-TB), an emerging challenge for TB control in the world, is described as resistant to rifampicin (RIF) and isoniazid (INH) drugs, with or without showing resistance to other first-line drugs [4]. Extensive drug resistance TB (XDR-TB) is also described as resistant to RIF, plus any fluoroquinolone, and at least one additional drug like linezolid, or bedaquiline [1, 5]. Globally, in 2019, roughly 500,000 reported new cases of RIF resistance, of which 78% had MDR-TB [3]. The RIF, the most potent first-line drug, accounted for 3.3% of new cases, and 17.7% of previously treated TB patients had MDR/RR-TB. In 2019, 6.2% of MDR-TB cases were estimated to have XDR-TB [3].

Ethiopia is among the highest TB, MDR-TB, and TB-HIV burden countries worldwide [3]. However, due to representative study limitations, the extent and distribution of drug resistance TB in TBLN cases in Ethiopia have yet to be known. The line probe assay (Genotype MTBDR assay) for the simultaneous detection of INH and RIF resistance is a recently developed method to assess the drug sensitivity of TB from clinical specimens and culture isolates of *Mycobacterium tuberculosis* complex (MTBC) [6]. The GenoType MTBDR*plus* assay detects resistance to RIF and INH in cultured samples based on detecting the most common mutations in the *rpoB* and *katG* genes, respectively. In addition, MTBDR*sl* assay detects specific gene mutations associated with fluoroquinolone (FLQ) resistance (*gyrA* and *gyrB* genes) and second-line injectable drugs (SLIDs) (*rrs* and *eis* genes) in the MTBC [7, 8].

According to current genomic study evidence, the MTBC is made up of five human-adaptable lineages (L1-4 and L7) that reflect MTB in a narrower sense, two other human-adaptable lineages (L5-6) that are historically referred to as *Mycobacterium africanum*, and at least nine animal-adapted lineages [9]. MTBC contains organisms commonly found in animals but can potentially spread as zoonotic diseases. Among the MTBC lineages, lineage 2, lineage 3, and lineage 4 are considered new lineages, while lineages 1, 5, and 6 are deemed ancient lineages. Lineage 7 is a premodern lineage phylogenetically located between ancient and modern lineages [10, 11]. In Ethiopia, both current and ancient lineages exist. Modern lineages (L3 and L4) are the most prevalent types [12]. Moreover, lineage 2 (Beijing strain), which is rarely detected in Ethiopia, has been linked to a high rate of drug resistance [13, 14].

The global emergence of drug-resistant strains through genetic diversity includes TB. Therefore, it is helpful for epidemiological studies to assess drug resistance caused by gene mutations in MTB and their strain diversity, which is spread out in a given geographical area. There have been many studies in Ethiopia to evaluate the pattern of drug resistance and the genetic diversity of MTB isolates from PTB patients. However, there needs to be more information available regarding TBLN studies. Considering this, this study is aimed at assessing drug resistance caused by gene mutations, which currently play a role in the global emergence of resistant strains to TB drugs through genetic diversity, and at investigating variations among MTBC strains in TBLN patients Addis Ababa, Ethiopia.

## 2. Materials and Methods

### 2.1. Study Area, Period, and Settings

This study was conducted at the All-African Leprosy and Tuberculosis Rehabilitation and Training Center (ALERT) and St. Peter TB Specialized Referral Hospitals from January 01 to April 2020 in Addis Ababa, Ethiopia. ALERT Hospital serves as a referral facility for skin diseases and reports 25–40 TB lymphadenitis suspected cases per week. The hospital also provides a pathology laboratory diagnostic service linked to Armauer Hansen Research Institute (AHRI). The St. Peter TB specialized referral hospital is a referral TB hospital from all parts of the country.

### 2.2. Study Design

A cross-sectional study of clinically suspected TBLN cases was conducted to determine the variations among MTB strains and drug-resistant patterns caused by gene mutations. This study included all TBLN-suspected patients who visited the study area during the specific period.

### 2.3. Sampling Procedure and Fine Needle Aspiration (FNA)

Consecutive sampling techniques were used to recruit 218 study participants, and approximately 50 *μ*L FNA samples were collected from the study participants. An experienced pathologist performed the standard FNA procedure [15]. FNA samples were collected from enlarged nodes using a 21-gauge needle after the overlying lymph node skin was disinfected with 70% ethanol, as explained elsewhere [16].

### 2.4. Laboratory Methods

#### 2.4.1. Culture Technique

The culture was performed for a total recruited of 218 FNAC samples on egg-based Lowenstein-Jensen-glycerol media with FNA fluid and incubated at 37°C for eight weeks, following the standard procedure. The inoculated culture was observed weekly for the presence of typical mycobacterial colonies. In addition, the Ziehl-Neelsen staining was used to confirm the acid fastness of all positive LJ cultures.

Ziehl-Neelsen staining was used to confirm the acid fastness of all positive LJ cultures with a dry, buff, wrinkled surface, rough raised, nonpigmented, and cream-colored colonies. No growth across the entire LJ media within eight weeks was recorded as culture-negative [17].

### 2.5. Region of Difference 9- (RD9-) Based Polymerase Chain Reaction

Heat-killed culture isolates were used for polymerase chain reaction- (PCR-) based deletion typing. The existence or absence of RD9 was used to distinguish MTB from other MTBC species using the following primers: RD9 flank F (5′-GTG TAG GTC AGC CCC ATC C-3′), RD9 intR (5′-CTG GAC CTC GAT GAC CAC TC-3′), and RD9 flank R (5′-GCC CAA CAG CTC GAC ATC-3′). PCR amplification was performed in a thermal cycler following standard procedure [18]. The cycling conditions were 10 minutes of enzyme activation at 95°C followed by 1 minute of denaturation at 95°C, 0.5 min of annealing at 55°C, 2 min of extension at 72°C, involving a total of 35 cycles, and final elongation at 72°C for 10 min. The product was electrophoresed in a 1.5 percent agarose gel with 1× TAE (Tris-acetate-EDTA) buffer. For gel electrophoresis, a 1 : 10 ratio of ethidium bromide, a 100 base pair DNA ladder, and orange 6× loading dye were used, and the results were visualized using a transilluminator (Bio-Rad Laboratories Inc.). A band size of 396 base pairs was considered positive for MTB detection [18, 19].

### 2.6. Line Probe Assay (LPA) GenoType MTBDR*plus* Test

Heat-killed bacterial colonies grown on LJ media were used for the GenoType MTBDR*plus* assay (Hain Lifescience, Nehren, GmbH, Germany). The bacterial colonies were collected with an inoculation loop, suspended in 100 *μ*L of lysis buffer (A-LYS) and heated at 95°C for 5 minutes to inactivate the vegetative bacilli. After a brief spinning down, an additional 100 *μ*L of neutralization (A-NB) buffer was added. Following 5 minutes of spinning the supernatant, DNA was used for PCR amplification. The master mix was prepared according to the manufacturer's instructions. The amplification profiles included 15 min of denaturation at 95°C, followed by 1 cycle of 30 sec at 95°C and 2 min at 65°C, followed by an additional 10 cycles of 25 sec at 95°C, 40 sec at 50°C, and 40 sec at 70°C, for a total of 20 cycles, and then an 8-minute extension at 70°C in 1 cycle. A TwinCubator (Hain Lifescience, Nehren, GmbH, Germany) was used for hybridization and detection [20].

### 2.7. Spoligotyping Technique

According to standard procedures, the isolates confirmed as MTB using the RD9 deletion test were further characterized with spoligotyping [21]. The thermal cycler was used to amplify the direct repeat (DR) region using oligonucleotides and two biotin-labeled primers, DRa: (5′GGT TTT GGG TCT GAC GAC3′) and DRb: (5′ CCG AGA GGG GAC GGA AAC3′). The reaction product was amplified using PCR. The target sequence was hybridized into 43 immobilized oligonucleotides, each corresponding of to one of the direct repeat (DR) locus' unique spacer DNA sequences. Following hybridization, the DNA was detected using enhanced chemiluminescence and X-ray film exposure as directed by the manufacturer.

In the most recent TB insight (http://tbinsight.cs.rpi.edu/run_tb_lineage.html) and SITVIT2 (http://www.pasteurguadeloupe.fr:8081/SITVIT2/) databases, the hybridization patterns were encoded into octal and binary formats and compared to previously reported strains [21, 22].

### 2.8. Data Analysis

Statistical analysis was done using the STATA 15 version (Stata Corporation, College Station, TX). Sociodemographic information, clinical data, and laboratory results are entered into the RED*cap* secure web-based database. In addition, the Fisher exact test was used to determine if there was a correlation between drug resistance caused by gene mutations and a particular MTB lineage and assess whether the difference between the values obtained was significant. All statistical tests were significant if the two-sided *p* value was less than 0.05.

### 2.9. Ethical Consideration

**Figure 1:**
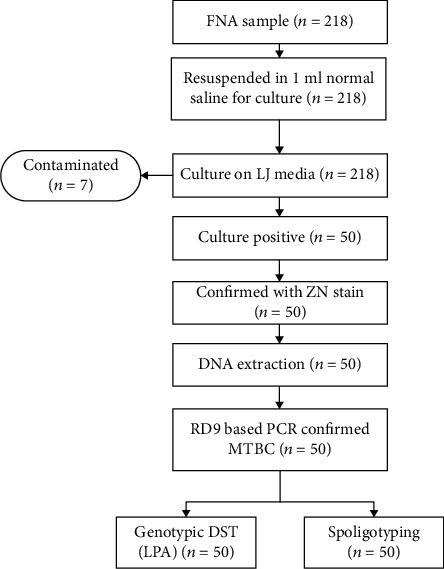
Flowchart of the study procedure.

The study was conducted after obtaining ethical approval from the Department of Medical Laboratory Science at Addis Ababa University College of Health Sciences (AAU-CHS) and the ALERT/AHRI ethical review committee (AAERC). The purpose of the study was explained to the study participants, and informed consent was obtained. Guardians or parents provided written consent for children. Assent was obtained from older children in addition to parental/guardian consent. Flow chart of the Study Procedure (Figure 1).

## 3. Results

### 3.1. Sociodemographic Characteristics of Study Participants

The mean age of the study participants was 29 (+/-14.45 SD) years and ranged from 5 months to 76 years, with a median age of 28 IQR (20–38). Most of the study participants, 52.3% (114/218), were 21 to 40. More than half were females, 61.9% (135/218) (male to female ratio of 1 : 2). Forty-one percent of the participants (91/21) were married, and 29.4% (64/218) were single. Two-thirds of the participants, 67.9% (148/218), came from urban areas. In this study, 19.7% (43/218) of participants were unemployed. Overall, the age, sex, marital status, living area, occupation, and educational status of the study participants were all found to have no statistical association.

### 3.2. Clinical Status of Study Participants

Of the 218 clinically confirmed TB lymphadenitis patients, 35.3% (77/218) of the study participants had previous antituberculosis treatment exposure. Based on the study participants' prior history of treatment exposure before enrollment, 83.3% (40/48) had completed their course of drug intake, while 16.7% (8/48) had discontinued anti-TB drugs. Sixteen percent of participants (35/218), 53.2% (116/218), 80.7% (176/218), and 41.7% (91/218) had a history of contact with known TB patients, a history of taking raw milk, no history of BCG vaccination, and living in the same household with livestock, respectively. In addition, all 218 study participants agreed to be tested for HIV, and 27.1% (59/218) of them were positive. Of the 59 confirmed HIV cases, 72.8% (43/59) had a previous history of anti-HIV treatment. In this study, night sweating, poor appetite, cough, previous contact with TB patients, history of raw milk utilization, living in the same household, intake, history of any antituberculosis treatment, HIV status, and BCG immunization were all found to have no statistical association.

### 3.3. Distribution of Affected Lymph Nodes

Of the 218 study participants, the most enlarged lymph nodes were unilateral left-side 42.2% (92/218) and right-side 40.8% (89/218) located lymphadenopathy. On the other hand, posterior and anterior cervical lymphadenopathy accounted for 25.2% (55/218) and 39.9% (87/218), respectively. It is followed by supraclavicular 11.5% (25/218) and axillary 10.1% (22/218) (Table 1). Of the total recruited FNA samples, 3.2% (7/218) were contaminated during incubation. The visual characteristics of the media, such as a color change from light green to blue, liquefaction, cottony growth, and breaking of the entire media, were all considered contaminated culture. The yield of culture positivity was relatively biting higher in samples collected from bilateral lymph node locations 34.3% (12/35), followed by unilateral right-sided 21.6% (19/88) and left-sided 21.8% (19/87) of clinically suspected patients.

### 3.4. Genetic Diversity and Family Pattern of the Bacterial Isolates

**Figure 2:**
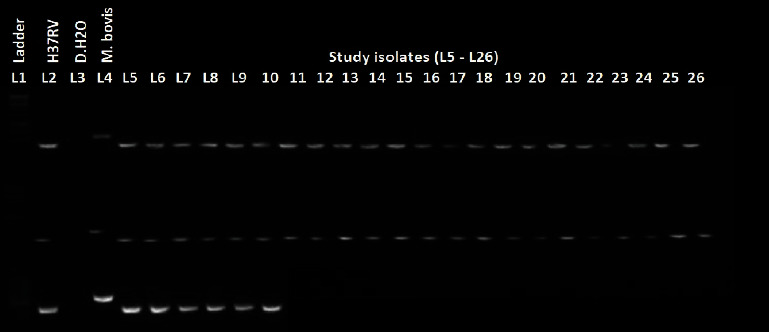
Region of difference 9 (RD-9) of *M. tuberculosis* strains isolated from TBLN patients from ALERT and St. Peter Specialized Hospital Addis Ababa, Ethiopia (2020).

This study analyzed 218 cases of suspected tuberculous lymphadenitis. Because of contamination of FNA samples during inoculation of LJ media incubation, seven cases were excluded from the final study. Fifty or 23.7% (50/211) isolates were cultured positively and harvested from LJ media. Among all those culture-positive isolates, 100% (50/50) were confirmed as MTB by PCR-based RD9 deletion typing (Figure 2).

Their spoligotyping consisted of 50 distinct patterns according to the websites of the TB insight and SITVIT2 databases. In cluster analysis, fifty isolates were sorted into 11 clusters based on spoligotyping patterns. Of the predominant spoligotype isolates, 62% (31/50) were previously known in the international database, and 38% (19/50) were orphan/newly found patterns. Based on their international spoligotype (SIT) classification, the dominant one was SIT-53 22% (11/50), SIT-37 16% (8/50), SIT-149 10% (5/50), and the rest was contributed by 2% (1/50). Using the TB insight and SITVIT2 websites, the distribution of their lineages was observed to fall into Euro-American Lineage (L4) for 78% (39/50), 18% (9/50) East African Indian lineage (L3), followed by 6% (3/50), and Indo-Oceanic Lineage (L1).

**Figure 3:**
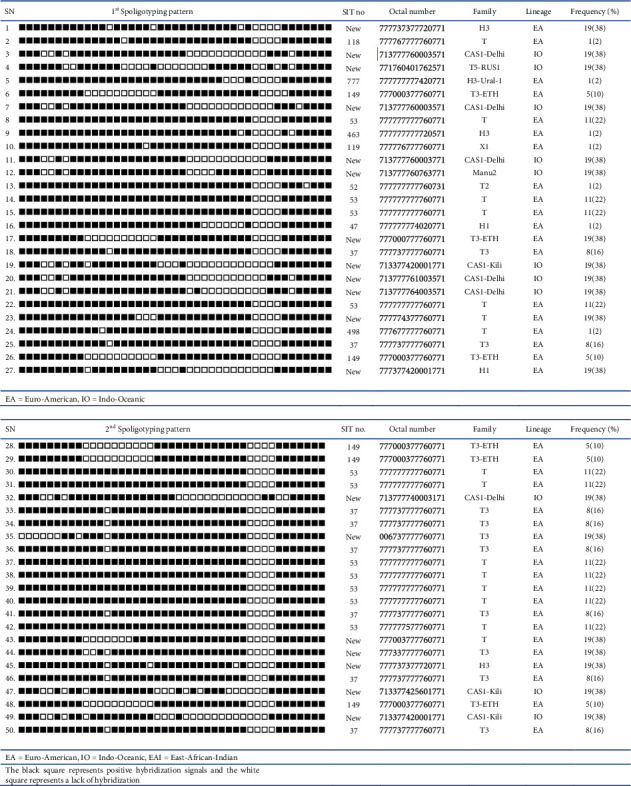
Spoligotype pattern of *M. tuberculosis* strains isolated from TBLN patients from ALERT and St. Peter Specialized Hospital Addis Ababa, Ethiopia (2020).

Based on (L4) family categories, 38.4% (15/39) belonged to Euro-American (T), 25.6% (10/39) to Euro-American (T3), 15.3% (6/39) to Euro-American (T3-ETH), 7.7% (3/39) to Euro-American (H3), and the rest to Euro-American (H3-Ural-1), Euro-American (X1), and Euro-American (T2). Among those of L1, each consists of Indo-Oceanic (CAS-Delhi) 54.5% (6/11), (CAS1-Kili) 27.2% (3/11), and each for (T5-RUS1) and (Manu2) (Figure 3).

### 3.5. Drug Susceptibility Profile of the Isolates

First-line antituberculosis drugs (RIF and INH) were tested for genotypic drug susceptibility, followed by second-line genotypic DST for fifty TB isolates.

Fourteen percent (7/50) of the isolates were resistant to any drug evaluated (INH and RIF). Any resistance to any single drug was identified as 6% (3/50) with RIF, followed by 14% (7/50) in the case of INH (Table 2).

Only the INH gene mutation caused by *katG* in the region interrogated at codon 315 in the S315T1 amino acid sequence resulted in the highest proportion, 8% (4/50) of mono drug-resistant isolates. On the other hand, a combined (INH and RIF) drug-resistant was observed in 6% (3/50). Their gene mutations were for *katG* and *rpoB* in the interrogated region at codon 315 with a change in the amino acid sequence of S315T1 and codon 530–533 with a difference in the amino acid sequence of S531L (S450L), respectively.

This study detected six percent (3/50) of MDR/RR-TB (Table 3). For those with first-line MDR resistant to genotypic TB drugs (both INH and RIF resistant) isolates, genotypic second-line (FLQ, KAN/AMK/CAP, KAN/CAP/VIO, KAN.AMK/CAP/VIO, low-level KAN) DST was done. Fortunately, no resistance was detected to the second-line genotypic DST. Two of the three MDR/RR-TB isolates were seen in HIV-positive patients.

### 3.6. Drug Resistance Caused by a Gene Mutation in *M. tuberculosis* Lineages

The genotypic strain variance of multidrug resistance was observed in 18.2% CAS1-Delhi (lineage 3) and 9% T3-ETH (lineage 4) strains. However, the association between anti-TB drug resistance caused by gene mutation and major MTB lineages was not statistically significant (Fisher's exact test: 0.118; *p* > 0.05).

Among those strains with 3 MDR/RR-TB, isolates with drug resistance caused by gene mutation, SIT498, SIT53, and orphan SITs showed resistance to the gene mutation of *katG*, whereas SIT149 and orphan were shown for *rpoB* gene mutations. The drug resistance caused by gene mutations in INH (*katG*) and RIF (*rpoB*) was observed in drug-resistant-associated gene loci. Four isolates tested resistant to the INH drug of the *katG* gene only with gene deletion in *katG*/WT and gene insertion in MUT1 (S315T1 change in amino acid sequences) have shown a mutation conferring INH resistance. No resistance to the *inhA* gene was observed. The gene deletion of the *rpoB*/WT8 mutation and the hybridization of rpoB/MUT3 (S531L change in amino acid sequences) were identified in two rifampicin-resistant MTB isolates spoligotype international type SIT149 and orphan strains. In contrast, the gene deletion in the *rpoB*/WT7 gene with the corresponding hybridization of *rpoB*/MUT1 (H526Y change in amino acids) was identified in one of the rifampicin resistance MTB isolates in the spoligotype international type of orphan strain (Table 3).

## 4. Discussion

In the present study, 86% (43/50) of the MTB isolates were sensitive to first-line anti-TB drugs. Fourteen percent of the analyzed strains resisted resistant to at least one first-line antituberculosis treatment. Mutations in genes were also observed in the evaluated MDR-associated gene loci in *katG* and *rpoB*. Four isolates tested resistant to the INH drug of the *katG* gene only with gene deletion in *katG*/WT and gene insertion in MUT1 (S315T1 change in amino acid sequences) conferring INH resistance. No *inhA* drug resistance-associated gene mutation was observed in this study. Several recent studies have looked at INH-resistant isolates with gene mutations in the *inhA* region of the promoter, which were found at a 0.8%, 10–12% frequency of *inhA* (without *katG*) mutation in Ethiopia [23–25], and somewhat higher than 30.8% and 43%, respectively [26, 27]. This shows that it may also play a limited role in the progression of ethionamide resistance [28], since it shares the same target in the mechanism of action and that administering high doses of INH to MDR-TB patients may have minimal effect. We also found the highest proportion of 4/7, 57% (4/7) caused by the *katG* gene mutation in the region interrogated at codon 315 and the region of the S315T1 insertion in INH-resistant MTB isolate, compared to 67% [26] and 100% in similar studies [29–31].

In the case of RIF, we also identified a mutation in the region interrogated at codon 530–533 with a change in the amino acid sequence of S531L insertion. This was most frequently reported in INH, and RIF drug-resistant MTB isolates from TBLN isolates in Ethiopia [26] and pulmonary TB [27, 32, 33]. In addition, we have identified the gene deletion in the *rpoB*/WT7 gene with the corresponding hybridization of *rpoB*/MUT1 in the H526Y amino acid change in the RIF resistance MTB isolates in this study. Our findings reported infrequent RIF drug resistance caused by gene mutations from TBLN isolates [26] and pulmonary TB cases in Addis Ababa [24, 27].

Compared to the same study, the result is higher than the prevalence rates revealed in TB lymphadenitis drug-resistant in Ethiopia [34] and lower than the rate reported in India [35]. This study found 6% of MDR/RR-TB lymphadenitis patients in three TB lymphadenitis patients. A recent report conducted in Ethiopia [36] indicated 1.4% of MDR-TB in new and previously treated TBLN patients and 0% [37] from Addis Ababa, whereas 1.6% reported from India [35]. The INH mono-resistance was 8% (4/50) in the present investigation, which is higher than the reports from Ethiopia, 3.6% [36].

In the current study, two MDR/RR cases were positive for HIV, and INH mono-resistance was more associated with HIV patients. Because the study sites were referral hospitals, particularly St. Peter for TB cases, the patients came from all over the country to be treated after different antibiotic treatment trials, which could explain why they did not respond to the first-line anti-TB drugs. INH mono-resistance is the initial move towards antituberculosis drug resistance to tranquilize opposition, and it is the common pathway for the advancement of MDR-TB [38].

The 50 isolates were further characterized by RD9 deletion-based PCR followed by spoligotyping [30, 39], and no *M*. *bovis* was found. This finding is consistent with studies conducted in various parts of Ethiopia [30, 39–41]. The absence of *M*. *bovis* as a causative agent of TBLN in patients may suggest that bovine TB may play a minor role in humans. It is also found in various MTB strains, including 11 different SIT and orphan/new strains. Most of the isolates (78%) belonged to the Euro-American lineage (L4), followed by East African Indian (L3), 18%. A recent study in Addis Ababa, Ethiopia (geographically similar to our study location) reported that 63.3% of the isolates were Euro-American, and 58.3% were Indo-Oceanic [39]. Studies from Northern Ethiopia (Dessie) reported that 57.1% are Euro-American, 28.6% are Indo-Oceanic, and 14.3% are East-African-Indian [30]. This may indicate that the Euro-American lineages are more widely distributed and predominant than all combined lineages.

According to the SITVIT2 and TB insight database, the spoligotyping international typing (SIT) numbers, the most prevalent shared types in the present study were new/orphan strains (38%), SIT53 (22%), SIT37 (16%), and followed by SIT149 (10%). These findings share similarities and differences with previous Ethiopian reports, with the dominant strain of SIT149 followed by SIT53 and SIT26 [39], and NEW strains at a higher rate following different SIT numbers [30]. Furthermore, SIT53 and orphan strains were the leading strains in Addis Ababa, Ethiopia [30, 39].

There needs to be more information regarding the drug resistance pattern of EPTB, especially in high-burden countries like Ethiopia. This is believed to be the difficulty of a limited number of laboratories in the country having the facilities to perform culture and drug susceptibility testing (DST) for MTB from extrapulmonary specimens. In addition, even the epidemiology of DRTB needs to be better understood in Ethiopia [42]. Because of these issues, we highly recommend further detailed.

## 5. Conclusion

This study found 3 MDR/RR-TB cases and heterogeneous strains of MTB among TBLN patients. The great extent of INH mono-resistance in HIV patients is a critical risk for the potential development of MDR-TB, as INH mono-resistance is a typical pathway to the occurrence of MDR-TB. The orphan and SIT53 (T) strains were the most common spoligotypes in the study population. In addition, a drug-resistant strain caused by mutation was detected among the clustered strains, indicating the transmission of clonal-resistant strains in the community.

The tool we used to characterize the MTB strains (spoligotyping) is prone to speciation evolution and has low-resolution power for cluster studies. However, this emphasizes the need for future research using a better discrimination tool to understand drug-resistant TB's transmission dynamics and must give special attention to TBLN and other extrapulmonary TB with a more integrated control strategy equal to pulmonary TB. Further similar studies should be conducted in this and other areas of Ethiopia to support comprehensive information because TBLN is not as directly infectious as pulmonary TB. Nevertheless, TBLN could be a reservoir of drug resistance unless patients are screened regularly.

## Figures and Tables

**Table 1:** Location and position of lymphadenopathy status of the study participant's data (*n* = 218) drowned from ALERT and St. Peter TB Specialized Hospital, Addis Ababa, Ethiopia 2020.

Variables	Clinically suspected TBLN *n* (%)	Culture negative *n* (%)	Culture positive *n* (%)
Location of lymph node			
Unilateral right-sided	89 (40.8%)	69 (78.4%)	19 (21.6%)
Unilateral left-sided	92 (42.2%)	68 (78.2%)	19 (21.8%)
Bilateral	36 (16.5%)	23 (65.7%)	12 (34.29%)
Generalized	1 (0.5%)	1 (100%)	0
Total	218 (100)	161 (76.3%)	50 (23.7%)
Position of lymph node			
Anterior cervical	87 (39.9%)	65 (75.6%)	21 (24.4%)
Posterior cervical	55 (25.2%)	40 (74.1%)	14 (25.9%)
Supra clavicular	25 (11.5%)	17 (73.9%)	6 (26.1%)
Axillary	22 (10.1%)	13 (65%)	7 (35%)
Mandibular	9 (4.1%)	6 (75%)	2 (25%)
Inguinal	16 (7.3%)	16 (100%)	0
Occipital	2 (1.0%)	2 (100%)	0
Femoral	1 (0.5%)	1 (100%)	0
Chest	1 (0.5%)	1 (100%)	0
Total	218 (100)	161 (76.3%)	50 (23.7%)

**Table 2:** Genotypic drug resistance frequency among M. tuberculosis strains of lymphadenitis TB patients at ALERT and St. Peter TB Specialized Hospital, Addis Ababa, Ethiopia.

Variables	First-line genotypic DST	Percentage (%)
Resistant to any single drug. Case = 7 (*n* = 50)		
Rifampicin	3	6%
Isoniazid	7	14%
Resistant to only a single drug. Case = 4 (*n* = 50)		
Isoniazid only	4	8%
Resistant to both drugs. Case = 3 (*n* = 50)		
Rifampicin + isoniazid	3	6%

**Table 3:** Common gene mutations observed in isoniazid and rifampicin-resistant MTB strains isolated from TB lymphadenitis patients in ALERT and St. Peter TB Specialized Hospital, Addis Ababa, Ethiopia.

TB drugs	Target gene	Gene mutation pattern (MUT/WT)	Change in amino acid sequences	INH/RIF mono-resistance (*n* = 4)	MDR-RR-TB (*n* = 3)	The pattern of drug resistance	SIT
INH	*inhA*	—	—	—	—	—	
	*KatG*	^−^WT/^+^MUT1	S315T1	3 (75%)	3 (100%)	INH-R	SIT498, SIT53, and orphan
		^−^WT/^+^MUT2	S315T2	1 (25%)	0	INH-R	Orphan

RIF	*rpoB*	-WT8/MUT3	S531L	0	2 (66.6%)	MDR	SIT149, orphan
		-WT7/MUT1	H526Y	0	1 (33.3%)	MDR	Orphan

^−^Gene deletion. ^+^Gene insertion. WT: wild type; MUT: mutation; INH-R: isoniazid resistance; SIT: spoligotype international type.

## Data Availability

All data are fully available without restriction.
